# Impact of Managerial Trustworthy Behavior on Employee Engagement: Mediating Role of Perceived Insider Status

**DOI:** 10.3389/fpsyg.2022.942697

**Published:** 2022-07-11

**Authors:** Defeng Liu, Haroon Bakari, Maharukh Niaz, Qianxiao Zhang, Imran Ahmed Shah

**Affiliations:** ^1^School of Economics and Finance, Xi’an Jiaotong University, Xi’an, China; ^2^Department of Business Administration, University of Sindh, Jamshoro, Pakistan; ^3^Department of Business Administration, ILMA University, Karachi, Pakistan

**Keywords:** managerial trustworthy behavior, perceived insider status, employee engagement, leadership, trust, trustworthiness, Pakistan

## Abstract

This study examines the impact of managerial trustworthy behavior on employees’ engagement and the mediating role of perceived insider status. This study has adopted an exploratory research design and positivist philosophy. The data are collected from 205 healthcare staff working in public sector hospitals in Pakistan through survey questionnaires, using a convenience sampling technique. Partial Least Square Structural equation modeling is used to analyze the data and test hypotheses. Results indicate that managerial trustworthy behavior relates positively to employee engagement. Perceived insider status mediates the relationship between managerial trustworthy behavior and employee engagement. The major limitation of this study is its cross-sectional design which limits the casualty. However, this study offers important insights regarding trust-building, engagement, and inclusion in the health sector. This study highlights the importance of trust-building among managers and employees. Managers who instill more trust in employees will garner more positive behavior. This study offers fresh insights into managers’ trustworthy behavior toward employees’ engagement and the employees’ perceived insider status within their organizations.

## Introduction

Individuals’ trust in healthcare systems and healthcare staff is key to the success of health sector organizations. Scholars have always emphasized developing interpersonal trust between managers and employees to foster positive behaviors (Yuan and Lee, 2022). Employee trust in leaders and managers is important for developing positive employee attitudes and behaviors (Dirks and Ferrin, 2002). Previous research has focused on leader-related factors to impact employee trust in leadership, such as transformational leadership, justice perceptions, perceived organizational support, and participative leadership, to name a few (Dirks and Ferrin, 2002). However, some important factors, such as the trustworthiness of leaders and managers themselves, have also been identified as an important source of not only developing positive behaviors in employees but also helping managers cope with negative aspects of work-life and retain confidence in employees (Korsgaard et al., 2002). For building a fruitful and agreeable relationship, the researchers found trust as the foundation of an effective relationship. Interpersonal trust is important for employee performance and organizational effectiveness (Tschannen-Moran and Hoy, 1998).

Social Exchange Theory can better explain interpersonal relationships between employees and managers (Gerstner and Day, 1997). Social exchange theory states a reciprocal relationship between managers and employees. If managerial behavior is perceived as trustworthy by employees, they will feel included in a core group of the leader and feel respected (Blau, 1964). Individuals engaged in the trusting behavior expect to do the same (Korsgaard et al., 2015).

Employees who experience trusting managers will show a greater level of employee engagement. Employee engagement involves employees’ physical, emotional, and cognitive energies (Kahn, 1990; Rich et al., 2010).

Employment relationships require trust between employees and managers and a sense of loyalty from both sides. Managers give the task to employees. The employees believe that they may be able to complete the task within the given time, and employees put extra effort into completing it; that is the trust that a manager has toward that employee. Such managerial acts ignite positive feelings among employees. Today’s job is so challenging for employees; they have much pressure. Trust is important to building a strong relationship with managers; it is a central attribute of managers’ and employees’ relationships (Korsgaard et al., 2015).

Recent research has outlined individuals’ trust in healthcare systems and physicians, which impacts the behaviors of healthcare workers (Yuan and Lee, 2022). However, research on how managerial trustworthiness and employee feeling of inclusion are important for employee engagement is less known. Therefore, based on the social exchange theory, this study aims to understand how managerial trustworthy behavior will be related to perceived insider status and subsequent employee engagement.

The previous research identified the perceived insider status role as a boundary condition between employee justice perceptions and employee expressions (Kim et al., 2019). This study extends previous understandings and constructs a model suggesting the mediating relation of insider status between managerial trustworthy behaviors and engagement of employees. This study will contribute to trust and inclusion in public health, an important aspect of public health management.

Therefore, this study answers a research question as to how managerial trustworthy behavior develops insider status and employee engagement. More specifically, this study aims to check the impact of managerial trustworthy behavior on employee engagement and use insider status as a mediator.

## Hypotheses Development

### Managerial Trustworthy Behavior

Managerial trustworthy behavior is defined as “volitional actions and interactions performed by managers that are necessary though insufficient to engender employees’ trust in them” (Whitener et al., 1998). Such managerial behavior is part of a greater economic and social exchange context. Managers build and maintain relationships with their employees by acknowledging their contributions by providing social and economic rewards. Employees then reciprocate these behaviors and trust managers. This trusting relationship is strengthened by other exchanges (Maxwell and Lévesque, 2014).

### Employee Engagement

Kahn (1990) presented the concept of personal engagement to indicate individuals’ psychological state where they utilize their personal resources and invest their positive energies, such as cognitive, physical, and emotional energies, to bring a difference in the workplace. Schaufeli et al. (2002) extended this concept of Kahn (1990) and related it to the workplace environment and defined engagement as “a positive, fulfilling, work-related state of mind that is characterized by vigor, dedication, and absorption” (p. 74). Both terms employee engagement and work engagement are used interchangeably. These reflect employees’ behavior directed at work characterized by positive cognitive, emotional, and physical energies immersed in a work setting, resulting in devotion, absorption, and dedication to work (Mackay et al., 2017).

### Managerial Trustworthy Behavior and Employee Engagement

Trust is important for a cordial relationship between leaders and followers (Griffith and Johnson, 2019). Recent studies suggested that trustworthy managers positively influence employee attitudes, behavior, workplace engagement, and work outcomes. It will create job satisfaction, job commitment, creativity, and engagement among the employees (Whitener et al., 1998; Colquitt and Rodell, 2011).

Employee engagement involves a particular person’s satisfaction with the eagerness for work (Harter et al., 2002). Managers who work with integrity and benevolence may give rise to the perception of justice and help employees cope with difficult and challenging times in their organizational lives (Cui and Jiao, 2019).

**Figure 1:**
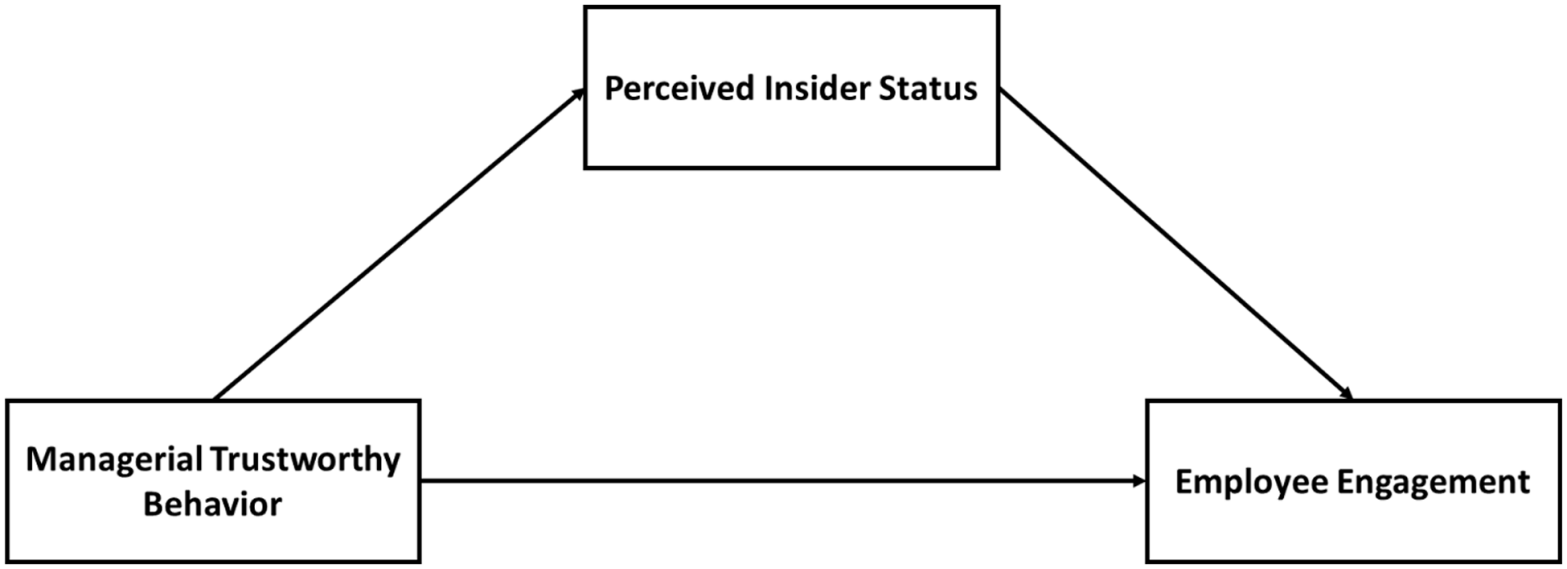
Theoretical framework.

If managers are sincere with employees and support them, employees, in return, will show a greater level of engagement. Being an effective leader is winning an employee’s trust. Recent research found that employees’ trust in leaders positively relates to employee engagement (Håvold et al., 2020). They also suggested that leaders’ trustworthiness is an important resource that is the outcome of leader-employee exchange and may result in employee engagement. Based on the above synthesis, this study hypothesizes that (Figure 1):

*H1*: Managerial trustworthy behavior is positively related to employee engagement.

### Perceived Insider Status

Perceived insider status (PIS) can be defined as how the individuals working in any organization perceive themselves as a part of the working organization (Stamper and Masterson, 2002, p. 876). Perceived insider status is concerned about the employee’s feelings about meeting the personal space, their perception of association toward the organization, and their belongingness is admitted in the organization (Masterson and Stamper, 2003). PIS also represents employees feeling of being part of an inner group. This membership is similar to having citizenship of an organization or a core group, making employees responsible and loyal to the organization and contributing with greater dedication and vigor (Graham, 1991).

### Managerial Trustworthy Behavior and Perceived Insider Status

Perceived insider status is the status of an employee within the organization where they perceive themselves as a part of an organization. If a manager trusts his employee and shows a good attitude toward them, involving them when making any decision, they feel like an insider of an organization.

PIS is also rooted in social exchange theory which presumes that people reciprocate behaviors, experiences, and values (Blau, 1964). Suppose managers interact with their employees with greater integrity, benevolence, and extra care. In that case, employees are likely to feel allied with leaders and the organization, thus developing a sense of insider status. Research suggests that trustworthy and transparent behavior will increase employee connectedness and perceived insider status (Colquitt and Rodell, 2011). Although some recent studies have found that perceived insider status as belongingness will increase employees’ trust in the organization, they also did not clarify the reciprocal relationship between trust and perceived insider status (Knapp et al., 2019). research also suggests that managerial feedback to employees positively develops their perception of being insiders (Chen et al., 2017). Based on the above synthesis, this study hypothesizes that:

*H2*: Managerial trustworthy behavior is positively and significantly related to perceived insider status.

### Perceived Insider Status and Employee Engagement

Scholars and practitioners are interested in building employee engagement because of its perceived positive impact on organizational effectiveness (Bakker and Leiter, 2010). Physical employee engagement refers to employees’ communication behavior in which employees raise their voices for their betterment (Dai and Chen, 2015). The perceived insider status is the employee’s position or rank in their organization. Employees’ perceived insider status is very important to let them know they are a significant part of the organization (Horng et al., 2015).

Research suggests that insider status motivates employees to engage in productive and cooperative activities (Blader and Tyler, 2009; Xu et al., 2021). Insider status perceptions increase employees’ likelihood of adopting and respecting organizational values such that they willingly strive hard to take more effort into work (Stamper and Masterson, 2002). Employees high on perceived insider status have a greater sense of achievement and feel that their contributions are and will be valued. They also feel that their contributions impact social and organizational wellbeing. Such self-concept also derives greater dedication, enthusiasm, loyalty, concentration, and vigor (Xu et al., 2021). Employee engagement is a positive attitude toward work characterized by employee willingness to invest more effort and being deeply absorbed in work (Schaufeli et al., 2002). Based on the above synthesis, the present study hypothesizes that:

*H3*: Perceived insider status is positively and significantly related to employee engagement.

### Mediating Role of Perceived Insider Status

Employee engagement reflects employees’ dedication and sincerity which is the outcome of organizational and managerial antecedents. Research suggests that public sector employees show greater engagement when supported by organizations and immediate supervisors (Jin and McDonald, 2016). Perceived support of the organization shows employees that organizations value their contribution to their work. Second, the superior’s support indicates that immediate supervisors trust them and share the same spirit of putting into teams (Jin and McDonald, 2016).

Research suggests that leaders’ roles and interpersonal communication are key to employee engagement (Bedarkar and Pandita, 2014). The group engagement model (Tyler and Blader, 2003) also suggests that when individuals experience managerial behaviors characterized by transparency, fairness, justice, and integrity, they are likely to identify with the managers based on respect. These feelings then facilitate employee psychological as well as behavioral engagement. This study thus hypothesizes that managerial trustworthy behavior will increase the insider status of employees. Additionally, perceived insider status will mediate the link between managerial trustworthy behavior and employee engagement. An experimental study from China found a positive impact of humble leadership on employee resilience, whereas perceived insider status mediated the link (Zhu et al., 2019). Another study found a negative association between leader narcissism and OCB. The link was mediated by perceived insider status (Wang et al., 2021). These studies confirm the nomological net of perceived insider status in managerial and leadership behaviors. Positive leadership interventions result in positive employee behaviors, and negative aspects of leadership and managerial conduct negatively impact employee perceptions.

In a longitudinal study, Xu et al. (2021) found perceived insider status as a mediator between justice perceptions and employee engagement. Based on the above synthesis, this study hypothesizes that:

*H4*: Perceived insider status mediates a positive relationship between managerial trustworthy behavior and employee engagement.

## Materials and Methods

### Participants and Procedure

Data were collected from healthcare staff of public hospitals in Sindh, Pakistan. The Convenience sampling technique was used to approach doctors, nurses, and paramedical staff of major public hospitals in Pakistan. After seeking proper permission, an online link to the survey was shared with the intended respondents. A total of 205 responses were received and subjected to data analysis.

### Measures

Managerial trustworthy behavior was measured using a 5-item scale developed by Whitener et al. (1998). Responses were recorded with the help of a 7-point Likert scale of 1 (strongly disagree) to 7 (strongly agree). Cronbach Alpha of the scale is 0.876.

Perceived insider status was measured using a scale developed by Stamper and Masterson (2002). Responses were recorded on the 7-point Likert scale ranging from 1 (strongly disagree) to 7 (strongly agree). Sample item includes “I feel I am an insider/outsider in my work organization” We also measure the scale reliability by computing Cronbach Alpha of 0.920.

We adopt the Utrecht work engagement scale (Schaufeli et al., 2016) to measure employee engagement. Nine items are included for measuring employee engagement by following 7- a point scale of 1 (never) to 7 (always). The sample item includes the “I am engaged at my work” item. We also measure the scale reliability by computing Cronbach Alpha of 0.842.

## Data Analysis

### Demographic Profile of Respondents

Table 1 indicates the sample distribution regarding age, gender, marital status, education level, and job position. Table 1 indicates that our sample consisted of 143 male respondents (70%), and the majority were young, having less than 34 years (153; 74%). The sample also consisted of most respondents from employees categories with non-managerial responsibilities (130; 63.4%) and having graduate degrees.

**Table 1:** Demographic profile.

	**Gender**	
	**Number**	**Percent**
Male	122	59.5
Female	83	39.5
Total	205	100
	**Age**	
	**Number**	**Percent (%)**
Below 18	7	3.4
18–24 years	85	41.5
25–34 years	61	29.8
35–44 years	31	15.1
45–54 years	19	9.3
55 above	2	1.0
Total	205	100
**Marital status**		
	**Number**	**Percent (%)**
Single	73	35.6
Married	132	64.4
Total	205	100
	**Job Position**	
	**Number**	**Percent (%)**
Nurses	78	38.0
Physicians	65	31.7
Paramedics	62	30.2
Total	205	100
	**Education**	
	**Number**	**Percent (%)**
14 years education	36	17.5
16 years education	109	53.2
18 years education	57	27.8
PHD	3	1.5
Total	205	100

### Model Estimation

Partial least square structural equation modeling tests the hypotheses (Hair et al., 2017). The major reason for selecting the PLS-SEM technique was that it is a causal-predictive technique that supports scholars in explaining and predicting the model (Shmueli et al., 2019). This study has analyzed and interpreted the data following the recommendations of Anderson and Gerbing (1988) and Hair et al. (2017). PLS-SEM is applied in a two-stage approach, i.e., estimating the measurement model and testing the structural model. In the analysis of the two-tail test, the values of external loadings should surpass the value of 0.708, the t-statistics should be higher than ±1.96, and 5% of the confidence interval will recommend indicator reliability at its sufficient level (Hair et al., 2014, 2020; Sarstedt et al., 2022). However, in some cases, if the outer loading value is 0.50, it is also acceptable (Hair et al., 2017).

### Measurement Model

The measurement model analyses construct reliability and convergent and discriminant validity. Construct reliability is estimated using two criteria, i.e., outer loadings and construct reliability (CR). Outer loading is estimated to know the relative contribution of each item to the construct (Harmann, 1976; Hair et al., 2017), and construct reliability (CR) reflects the ability of the construct to yield consistent results. It also reflects how indicators (items) of a construct estimate numerous aspects of a focal construct (Revicki, 2014). Convergent validity is measured using the average variance extracted. It reflects that all indicators of the focal construct are attributed to the same construct (Henseler et al., 2009). The last estimation in the measurement model is the analysis of the discriminant validity of the model. Discriminant validity refers to the condition where all constructs of the model that are thought to be different are different (Hair et al., 2017). In other words, the constructs that are conceptually different should empirically be proven as different (Henseler et al., 2009).

The reliability of outer loading must be greater than 0.50 (Hair et al., 2017) and, the composite reliability should be greater than 0.70 (Hair et al., 2017), the AVE values should be greater than 0.50 suggested by Hair et al. (2017).

**Figure 2:**
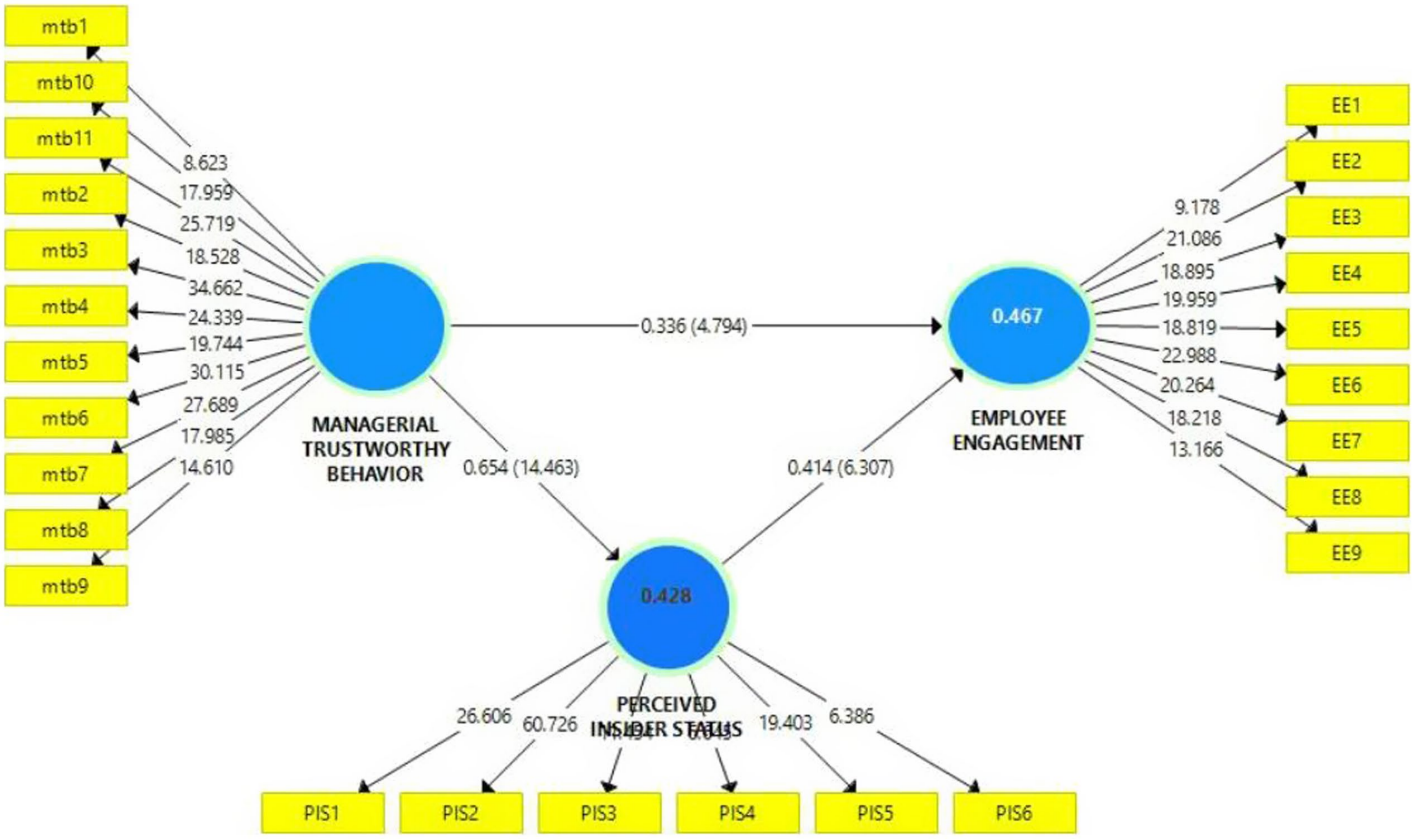
Structural model.

Table 2 shows the data results that the value of CR is greater than its cutoff value of 0.70 and the values of Average variance extracted are greater than its cutoff value of 0.50 (Figure 2).

**Table 2:** Results of reliability and validity.

**Latent variables**	**Items retained**	**Outer Loadings**	**CR**	**AVE**
**MTB**	MTB1	0.566	0.901	0.505
	MTB2	0.752		
	MTB3	0.831		
	MTB4	0.777		
	MTB5	0.751		
	MTB6	0.796		
	MTB7	0.782		
	MTB8	0.707		
	MTB9	0.694		
	MTB10	0.739		
	MTB11	0.804		
**PIS**	PIS1	0.842	0.933	0.560
	PIS2	0.892		
	PIS3	0.713		
	PIS4	0.576		
	PIS5	0.787		
	PIS6	0.556		
**EE**	EE1	0.534	0.875	0.546
	EE2	0.742		
	EE3	0.709		
	EE4	0.749		
	EE5	0.741		
	EE6	0.760		
	EE7	0.736		
	EE8	0.727		
	EE9	0.670		

MTB, managerial trustworthy behavior; PIS, perceived insider status; EE, employee engagement.

Table 3 shows the results of discriminant validity as per Fornell and Lacker criteria. Results indicate that squared root of AVE (values in bold) is greater than inter-construct correlations. Table 4 describes discriminant validity in terms of HTMT values. All the values are less than 0.85, HTMT scores that are <0.85 show that the discriminant validity is good, and if the values are >0.90, then this shows the discriminant validity is poor (Henseler et al., 2014; Hair et al., 2017; Table 4).

**Table 3:** Discriminant validity: Fornell–Larcker criterion.

Constructs	**MTB**	**PIS**	**EE**
MTB	**0.711**		
PIS	0.607	**0.748**	
EE	0.634	0.654	**0.739**

MTB, managerial trustworthy behavior; PIS, perceived insider status; EE, employee engagement.

**Table 4:** Discriminant validity.

	EE	MTB	PIS
**EE**			
**MTB**	0.666		
**PIS**	0.671	0.647	

MTB, Managerial trustworthy behavior; PIS, Perceived insider status; EE, Employee engagement.

### Structural Model

Once reliability and validity are established through measurement model analysis, the structural model is analyzed five-step approach suggested by Hair et al. (2017). This begins with checking for multicollinearity issues among predictors of the model. All VIF values are less than 3.3, thus indicating that the model is free from multicollinearity problems (Diamantopoulos and Siguaw, 2006).

Next, the beta coefficients were calculated using the bootstrapping method with 5,000 resampling to test hypotheses. Results are presented in Table 5. The first hypothesis was related to MTB’s positive and significant impact on EE. The result of this study reveals that the value of beta coefficients *β* = 0.338, *t* = 4.820 (>1.96), *p* < 0.05, and there is no zero in between the confidence interval CI (0.199; 0.477). The result shows that MTB has a significant and positive impact on EE, so the first hypothesis has been accepted.

**Table 5:** Significance and relevance of direct and indirect paths coefficients.

Path	***β***	**SD**	***T* Value**	**5.0%**	**95.0%**	***f*** ^**2**^	***Q*** ^**2**^	**VIF**	***R*** ^**2**^	**Supported**
**MTB** → **EE**	0.338	0.070	4.820	0.199	0.477	0.121	0.385	1.747	0.467	Yes
**MTB** → **PIS**	0.660	0.046	14.465	0.564	0.741	0.747	0.476	1.00	0.428	Yes
**PIS** → **EE**	0.417	0.066	6.390	0.284	0.537	0.184	0.387	1.747		Yes
**MTB** → **PIS** → **EE**	0.3414	0.065	5.253	0.215	0.475					Yes

MTB, managerial trustworthy behavior; PIS, perceived insider status; EE, employee engagement.

The second hypothesis was related to MTB’s positive and significant impact on PIS. The result of this study indicates that the value of beta coefficients *β* = 0.660, *t* = 14.465 (>1.96), and *p* < 0.05 are adequate, and there is no zero in between the confidence interval CI (0.564; 0.741). It reveals that MTB has a significant and positive impact on PIS, so the second hypothesis is also accepted.

The third hypothesis was related to the positive and significant impact of PIS on EE. The results also indicate the beta coefficients *β* = 0.417, *t* = 6.390 (>1.96), *p* < 0.05 are adequate, and there is not any zero in between the confidence interval CI (0.284; 0.537). The results reveal that PIS significantly and positively impacts EE, so the third hypothesis is accepted.

A fourth hypothesis related to mediation of perceived insider status (PIS) between MTB and EE. Results show that the beta coefficients of the indirect path are *β* = 0.3414, *t* = 5.253 (>1.96), *p* > 0.05, and there is no zero in between the confidence interval CI (0.215; 0.475). It shows that PIS mediates the positive effect of MTB on EE, So the fourth hypothesis is also accepted. After the analysis of beta coefficients, *t*-values, and confidence interval, R square is checked to see variance accounted for by predictors of the model in exogenous variables. It is revealed that predictors of the model account for 47 and 43% of employee engagement and perceived insider status, respectively. Next, values of *f*^2^ (effect size) were checked (Sullivan and Feinn, 2012) to see the relative relevance of each path. It is revealed in Table 5 that employee engagement is showing a medium effect size with managerial trustworthy behavior (*f*^2^ = 0.121) and perceived insider status (*f*^2^ = 0.184), respectively. At the same time, managerial trustworthy behavior shows a large effect size with perceived insider status (*f*^2^ = 0.747; Cohen, 1992).

Finally, using the blindfolding technique, the predictive relevance of the model is evaluated through the Q^2^ estimate. Results indicate that all Q^2^ values of endogenous variables of the study are greater than zero (0.217 and 0.203 for employee engagement and perceived insider status, respectively). These estimates show that the model is predictively relevant (Hair et al., 2017).

## Discussion

This section deals with the interpretation of the results of this study and discusses the research findings dependent on the literature and theory. Here we also discuss the study’s limitations and implications and the recommendations set for the future.

The main motive of this research has been set to test the impact of managerial trustworthy behavior on employee engagement and the mediating role of perceived insider status between managerial trustworthy behavior and employee engagement. To do this, we have reported a review of the literature where the first objective was to discuss the theory and model of the study, and the second objective was to test the model. We have collected data from various organizations to test the managerial trustworthy behavior on employee engagement in organizational settings. The timeline for collecting the present research data is 4–6 months by meeting people and collecting their feedback with survey questionnaires by personally visiting the employees of different organizations.

The conceptual model of this study consisted of three variables, i.e., managerial trustworthy behavior as the independent variable, employee engagement as a dependent variable, and perceived insider status as a mediating variable. The literature also recommended how these three constructs are interrelated, and linked with each other, suggested how the social exchange theory relates to this study and clarified the relation of the construct with the social exchange theory.

The social exchange theory (Blau, 1964) helps describe trust between supervisors and employees. Social exchange is a process of exchange. Employees of an organization are engaged with three types of social exchange to maintain relationships with the working organization. First, they have to maintain a relationship with their coworkers or colleagues. Second, they have to maintain relationships with their superiors or managers. Furthermore, the third, they have to maintain relationships with the working organization (Masterson et al., 2000).

Managerial trustworthy behavior influences employee engagement, i.e., coherent, communicative, behavioral, sentimental, and interactive energies invested by the employees (Shuck et al., 2017). Perceived insider status is the mediating role between managerial trustworthy behavior. PIS is the development of a relationship between an organization and employees. It is an employee’s perception of being part of an organization, and employees perceive themselves as insiders (Stamper and Masterson, 2002).

Our study has developed the theoretical framework to explain the impact of managerial trustworthy behavior on employee engagement. Managerial trustworthy behavior is the independent variable of our study, and employee engagement is a dependent variable. Perceived insider mediating between the independent and dependent variables of the study, where managerial trustworthy behavior is connected with employee engagement and perceived insider status links managerial trustworthy behavior and employee engagement. We have tested the hypotheses of all these variables presented in the study.

So in our study, all the hypotheses have been accepted: Our first hypothesis relates to managerial trustworthy behavior and employee engagement. There are positive and significant relationships among them. The previous study shows a positive and significant relationship between feeling trusted and employees (Rouzi and Wang, 2021).

Our second hypothesis relates to managerial trustworthy behavior and perceived insider status. According to our research, there is a positive and significant relationship between them. According to our findings in the previous study, there is a positive and significant relation between feeling trusted and perceived insider status.

A recent study that tested predictors of managerial trustworthiness and untrustworthiness found that managers need three things to do to develop trustworthiness. Communication, engagement and cognitive diversity (Tigre et al., 2022). Our study using social exchange theory focusing on communication between leaders and members has argued that managerial trustworthiness will positively be related to perceived insider status and employee engagement (Whitener et al., 1998; Korsgaard et al., 2002; Cui and Jiao, 2019).

### Limitations and Future Recommendations

There are some limitations to this research. Firstly, data were collected from multiple sectors. Future research should collect data from specific sectors to see specific effects for a particular sector. Secondly, we collected data from only a single source, i.e., further in future testing, there should be data collection from the managers. Thirdly, this data collection occurred in a cross-sectional design. Future research should conduct a longitudinal study to establish the causality between variables. This study tested perceived insider status as a mediator and did not include boundary variables. The managerial trustworthy variable may be perceived differently by people with different propensity levels to trust (Patent and Searle, 2019).

Therefore, future research may use the propensity to trust as a moderator between trustworthy behavior and perceived insider status. This study tested managerial trustworthy behavior and its impact on employees’ perceived insider status. However, future research may explore different interesting streams, such as testing the trustworthiness of employee behaviors (Korsgaard et al., 2002), its impact on managers’ responses toward employees, and whether employees’ trustworthiness develops managerial behaviors that promote other employee attitudes and behaviors.

This study found that managerial trustworthy behavior develops perceived insider status, which will develop employee engagement. A recent study found an association between inclusion, communication, engagement, and leader trustworthiness, which has suggested that trustworthiness is the function of these conditions. Such a finding implies that there might be the possibility of a reciprocal relationship between trustworthiness and insider status and engagement (Tigre et al., 2022). For example, unlike our results, future research may check the possibility that employee engagement and insider status may develop managerial trustworthiness. Previous research has argued the reciprocal relationship between justice and trustworthiness (Lance Frazier et al., 2010; Colquitt and Rodell, 2011).

### Implications

This study also offers some practical implications for managers and leaders of the organizations. Firstly, this study suggests that managers who display behaviors consistent with employees’ expectations and are reliable may develop trustworthiness in their behaviors and consequently develop connectedness with their subordinates. Employees who feel connected to their managers and feel like members of the inner or core group will show greater dedication, engagement, and vigor (Fernando et al., 2021).

Modern world managers wish to increase employee, behavioral, and psychological engagement (Xu et al., 2021). Managers of Pakistani organizations will benefit from this study and should adopt behaviors that enhance their trustworthiness.

## Conclusion

The study has examined the impact of managerial trustworthy behavior on employee engagement and mediating role of perceived insider status. This study has used social exchange theory to understand the underlying relationships between managerial trustworthiness and employee feeling of insider status. This study contributes to the literature on trust and insider status. Managers’ actions that make them trustworthy are important for their relationship with employees and their engagement in work.

## Data Availability Statement

The raw data supporting the conclusions of this article will be made available by the authors, upon request.

## Author Contributions

All authors listed have made a substantial, direct, and intellectual contribution to the work and approved it for publication.

## Conflict of Interest

The authors declare that the research was conducted in the absence of any commercial or financial relationships that could be construed as a potential conflict of interest.

The reviewer AH declared a past co-authorship with the author HB to the handling editor.

## Publisher’s Note

All claims expressed in this article are solely those of the authors and do not necessarily represent those of their affiliated organizations, or those of the publisher, the editors and the reviewers. Any product that may be evaluated in this article, or claim that may be made by its manufacturer, is not guaranteed or endorsed by the publisher.
